# Sunflower Plants as Bioindicators of Environmental Pollution with Lead (II) Ions

**DOI:** 10.3390/s90705040

**Published:** 2009-06-25

**Authors:** Olga Krystofova, Violetta Shestivska, Michaela Galiova, Karel Novotny, Jozef Kaiser, Josef Zehnalek, Petr Babula, Radka Opatrilova, Vojtech Adam, Rene Kizek

**Affiliations:** 1 Department of Chemistry and Biochemistry, Mendel University of Agriculture and Forestry, Zemedelska 1, CZ-613 00 Brno, Czech Republic; 2 Department of Plant Biology, Mendel University of Agriculture and Forestry, Zemedelska 1, CZ-613 00 Brno, Czech Republic; 3 Department of Chemistry, Faculty of Science, Masaryk University, Kotlarska 2, CZ-611 37 Brno, Czech Republic; 4 Institute of Physical Engineering, Faculty of Mechanical Engineering, Brno University of Technology, Technicka 2896/2, CZ-616 69 Brno, Czech Republic; 5 Department of Natural Drugs, Faculty of Pharmacy, University of Veterinary and Pharmaceutical Sciences, Palackeho 1-3, CZ-612 42 Brno, Czech Republic; 6 Department of Animal Nutrition and Forage Production, Faculty of Agronomy, Mendel University of Agriculture and Forestry, Zemedelska 1, CZ-613 00 Brno, Czech Republic

**Keywords:** phytoremediation, heavy metals, sunflower, lead ions, high performance liquid chromatography with electrochemical detection, spectrometry, laser induced breakdown spectroscopy

## Abstract

In this study, the influence of lead (II) ions on sunflower growth and biochemistry was investigated from various points of view. Sunflower plants were treated with 0, 10, 50, 100 and/or 500 μM Pb-EDTA for eight days. We observed alterations in growth in all experimental groups compared with non-treated control plants. Further we determined total content of proteins by a Bradford protein assay. By the eighth day of the experiment, total protein contents in all treated plants were much lower compared to control. Particularly noticeable was the loss of approx. 8 μg/mL or 15 μg/mL in shoots or roots of plants treated with 100 mM Pb-EDTA. We also focused our attention on the activity of alanine transaminase (ALT), aspartate transaminase (AST) and urease. Activity of the enzymes increased with increasing length of the treatment and applied concentration of lead (II) ions. This increase corresponds well with a higher metabolic activity of treated plants. Contents of cysteine, reduced glutathione (GSH), oxidized glutathione (GSSG) and phytochelatin 2 (PC2) were determined by high performance liquid chromatography with electrochemical detection. Cysteine content declined in roots of plants with the increasing time of treatment of plants with Pb-EDTA and the concentration of toxic substance. Moreover, we observed ten times higher content of cysteine in roots in comparison with shoots. The observed reduction of cysteine content probably relates with its utilization for biosynthesis of GSH and phytochelatins, because the content of GSH and PC2 was similar in roots and shoots and increased with increased treatment time and concentration of Pb-EDTA. Moreover, we observed oxidative stress caused by Pb-EDTA in roots where the GSSG/GSH ratio was about 0.66. In shoots, the oxidative stress was less distinctive, with a GSSG/GSH ratio 0.14. We also estimated the rate of phytochelatin biosynthesis from the slope of linear equations plotted with data measured in the particular experimental group. The highest rate was detected in roots treated with 100 μM of Pb-EDTA. To determine heavy metal ions many analytical instruments can be used, however, most of them are only able to quantify total content of the metals. This problem can be overcome using laser induced breakdown spectroscopy, because it is able to provide a high spatial-distribution of metal ions in different types of materials, including plant tissues. Data obtained were used to assemble 3D maps of Pb and Mg distribution. Distribution of these elements is concentrated around main vascular bundle of leaf, which means around midrib.

## 1. Introduction

**Figure 1.**
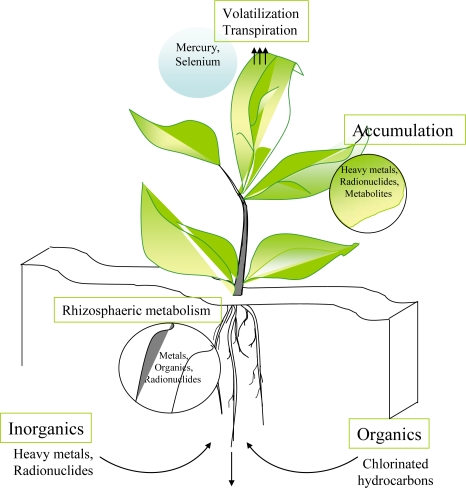
Phytoremediation can occur through a series of complex interactions between plants, microbes, and the soil, including accumulation, hyperaccumulation, exclusion, volatilization, and degradation of the target pollutant. Plants also stabilize mobile contaminated sediments by forming dense root mats inside soil.

Environmental remediation deals with the removal of contaminants from soil, groundwater, sediment, surface water etc. for the general protection of human health and the environment [1]. Remediation processes can be expensive, as the are mostly *ex-situ* methods involving excavation of impacted soils and subsequent treatment at the surface. Therefore new, efficient, inexpensive and non-environmentally disruptive technologies are still developing. One of the groups of such new technologies is called bioremediation. It involves the treatment of environmental problems through organisms. Microorganisms (e.g., *Desulfomonile*, *Clostridium*, *Pseudomonas*, *Acinetobacter*) and plants (e.g., *Betula*, *Populus*) are most commonly used for these purposes [2–7]. If plants are used, we call this process phytoremediation [1,8–10]. A range of processes mediated by plants are useful in treating environmental problems. Plants can chemically modify toxic substances as a direct result of plant metabolism (phytotransformation), can reduce the mobility of substances in the environment (phytostabilization) or uptake and concentrate substances from the environment into the plant biomass (phytoextraction). The scheme of various ways how to a plant metabolizes or deposit the pollutant is shown in Figure 1.

Most toxic substances (organic pollutants, heavy metals) come from anthropogenic activities such as mining, traffic, heavy industry, etc. [11–13]. Contrary to organic pollutants, heavy metals cannot be degraded or destroyed. To a small extent they enter our bodies via food, drinking water and air. As trace elements, some heavy metals (e.g., copper, selenium, zinc) are essential to maintain the metabolism of the human body. However, others such as cadmium, lead, and mercury are toxic at all. At higher concentrations both groups of heavy metals (toxic and essential) can lead to poisoning [13]. Heavy metals are also dangerous because they tend to bioaccumulate. Lead is one of the most dangerous and toxic heavy metals. Levels of lead in the environment are not stable and vary according to industrial production, urbanization, climate changes and many other factors [14]. The levels of lead in the environment vary between 4 and 20 mg/g of dust. Uncontaminated waters contain lead in concentrations ranging from 0.001 to 0.06 mg/L. In soils, levels of lead reach 5 to 30 mg per kg of soil. When lead is added into petrol as an additive, the highest lead levels are determined on the surfaces of leaves, from where lead enters the food chain, as well as soil or water. Lead is present in soils as salts in soluble as well as insoluble forms. Lead contamination in the soil is known to inhibit seed germination [15,16]. The inhibition of germination by exogenously supplied Pb^2+^ is a possible effect of interference with some important enzymes involved in the process. Photosynthesis is considered as one of the metabolic processes most sensitive to Pb^2+^ toxicity [17]. Closing of the stomata, disruption of the chloroplastic organization, change in the metabolites of photosynthesis and replacement of essential ions like magnesium are the main effects on photosynthesis of lead toxicity [14,18–21]. The metal has also been reported to inhibit photosynthesis in isolated chloroplasts. There have been also published data reporting on inhibition of enzymes crucial for nitrogen assimilation [14].

**Figure 2.**
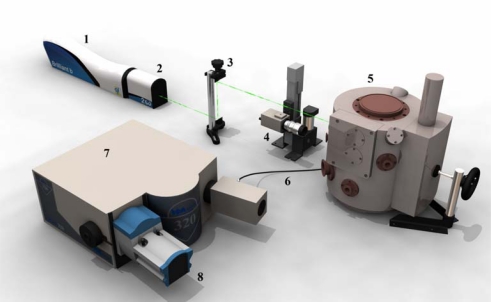
Experimental arrangement of LIBS: 1 – Nd:YAG laser, 2 – modulator of second harmonic frequency, 3 – periscope, 4 – CCD camera, 5 – ablation chamber, 6 – fibre optic system, 7 – monochromator, 8 – ICCD camera.

**Figure 3.**
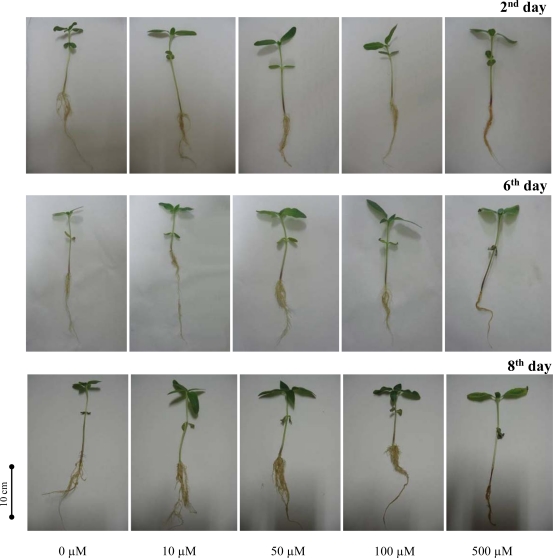
Pictures of sunflower plants in the second, sixth and eighth experimental day after Pb-EDTA application (0, 10, 50, 100 and 500 μM).

As we mentioned above for the particular example of lead ions, there are many mechanisms and pathways which can be affected by heavy metals [22–25]. Protective mechanisms of a plant cell against the toxic effects of heavy metals mainly involve synthesis of compounds rich in cysteine called phytochelatins. Their synthesis comes from the most abundant thiol – reduced glutathione. To detect these compounds many various methods and techniques have been employed [23,24,26–30], including sensors and biosensors [31–33]. However, uptake and transport as well as storage of heavy metals through plant tissues remain still unclear. To consider whether a specific plant species is able or not able to remediate the polluted environment, not only heavy metals content in the plant tissues, but also the distribution of such metal ions in the tissues must be analysed. Analytical methods and instruments for detection of lead (II) ions have been reviewed several times [34–38]. The diagnostic techniques enabling monitoring high spatial- and lateral-distribution of elements within different plant structures include mainly X-ray imaging methods [39–41]. X-ray microscopy and micro-radiography investigations usually make use of soft X-rays generated by plasma laser, microfocus X-ray sources and synchrotron radiation [42]. Although the X-ray radiation based methods are relatively high-cost and availability of the experimental apparatus is limited due to possibility of “*in-situ*” analysis only, it offers new aspects for studying the distribution of heavy metals. However, X-ray imaging methods are intensively investigated in our laboratories; recently we have been focusing also on the realization of spatially-resolved spectro-chemical analysis by utilizing laser-ablation based techniques. Laser induced breakdown spectroscopy (LIBS, Figure 2) is a type of atomic emission spectroscopy which utilises a highly energetic laser pulse as the excitation source and is able to provide high spatial-distribution of metal ions in different types of materials [43–45]. The character of the ablative process depends on the features of the laser used (wavelength, pulse duration, power and energetic profile of the rays), surrounding atmosphere and the features of the sample itself (matrix, absorption characteristics, its structure) [46]. LIBS method is one of analytical instruments which makes qualitative and quantitative analysis and also monitoring of element distribution in different types of samples possible. The main advantage of this method is that it requires no, or minimal sample pre-treatment and enables multi-elemental analysis with high three-dimensional resolution. A limiting factor is especially the diameter of laser ray. In this study, the influence of lead (II) ions on sunflower plants (Figure 3) was investigated from various points of view. We aimed our study at common growth parameters, morphological changes, total protein content, activity of certain enzymes, level of stress induced thiols and spatial distribution of lead.

## 2. Results and Discussion

### 2.1. Morphological changes

**Figure 4.**
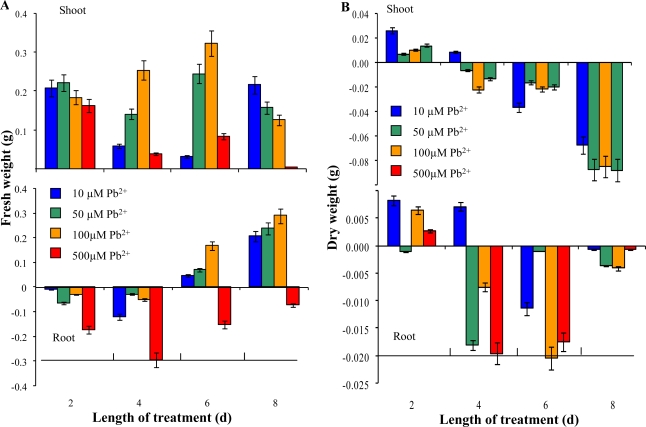
Changes of (A) fresh and (B) dry weights of sunflower plants exposed to Pb-EDTA. Dry mass was obtained by drying to the constant weight at 105 °C in the oven. All data were obtained by subtraction from control plants. The experiment was carried in triplicates.

Lead is a poisonous metal that has many adverse effects on plants and animals. Sunflower plants were treated with 0, 10, 50, 100 and/or 500 μM Pb-EDTA for eight days. We observed alterations in growth in all experimental groups compared with non-treated control plants. Plants exposed to lead (II) ions grew faster in comparison with control plants, except for the highest applied concentration. This phenomenon probably relates to the stimulatory effects of the presence of Pb-EDTA, because control plants were cultivated in distilled water only, where no other nutrients are present. In addition we observed chlorosis on plants treated with the highest concentration (Figure 3). When we compared the fresh weight of plants treated with lead (II) ions with the non-treated experimental group, it was possible to clearly notice the effect of applied Pb-EDTA concentration on the aerial parts of plants, except for the highest applied concentration (Figure 4 A).

Nevertheless, the adverse effect of lead (II) ions is shown on dependence of dry weight on length of the treatment and applied concentration (Figure 3B). Determined change is probably connected with increased water uptake of plants exposed to stress caused by heavy metals. This hypothesis is supported by results reporting on nuclear magnetic resonance analysis of early somatic embryos clusters [29]. In the case of change in fresh weight of roots, increases by the sixth and eighth day were detected, except for the highest applied concentration. Dry weight of roots decreased, except on the second day of the treatment for all experimental groups (Figure 4 A,B).

### 2.2. Total protein content

**Figure 5.**
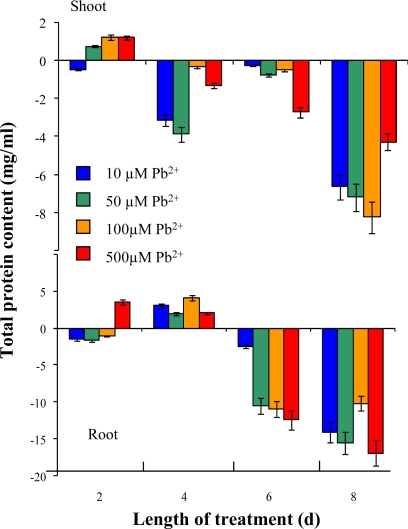
Changes in total protein content in sunflower plants exposed to Pb-EDTA. All data were obtained by subtraction from control plants. The experiments were carried out in triplicate.

Heavy metals taken up by plants or induce stress reactions, which manifest as enhancements of the levels of certain molecules. Firstly, the level of mRNA is enhanced with subsequent changes in protein profile. Therefore, we determined total content of proteins by a Bradford protein assay. Total content of proteins slightly increased in both aerial parts and roots in the second day. From the fourth day of the treatment, a decrease of total content of proteins occurred. In eighth day of the experiment this loss was approx. 8 μg/mL or 15 μg/mL in shoots or roots of plants treated with 100 mM Pb-EDTA (Figure 5). Total content of proteins is dramatically reduced thanks to the heavy metal ions. This trend matches well with the dry weight dependence (Figures 4 and 5).

### 2.3. Determination of plant enzymes’ activity

**Figure 6.**
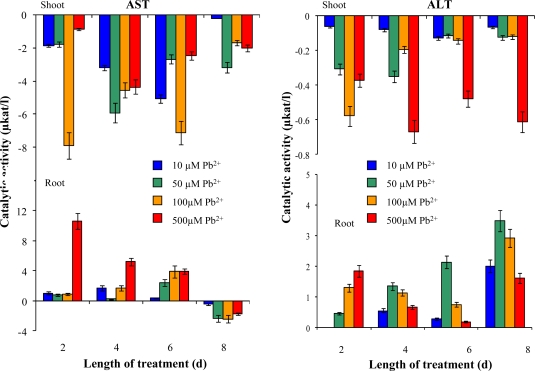
Changes of AST and ALT activities in sunflower plants exposed to Pb-EDTA. All data were obtained by subtraction from control plants. The experiment was carried in triplicates.

There is still not much available information about the significance of some commonly analyzed enzymes as markers of stress reactions in plants. In several papers, we have demonstrated that some enzymes (such as aminotransferases or urease) can participate in plant stress reactions [22,26,47–51]. Thus, we focused our attention on the activity of alanine transaminase (ALT), aspartate transaminase (AST) and urease. Transaminases catalyze the transfer of the amino groups of amino acids to 2-oxo-acids. In plants, transaminases participate very effectively in transformations of nitrogen compounds. They are important for the synthesis of amino acids from oxo-acids in the citrate cycle and for other crucial biochemical pathways. They also play key roles in the synthesis of secondary metabolites as well as chlorophyll. In roots AST and ALT activities were increased during the experiments in comparison to control plants (Figure 6). This increase corresponds well with the higher metabolic activity. Urease activity was enhanced in aerial plant parts as well as in roots with increasing length of exposition and applied concentration slightly (data not shown).

### 2.4. Content of low molecular mass thiols

**Figure 7.**
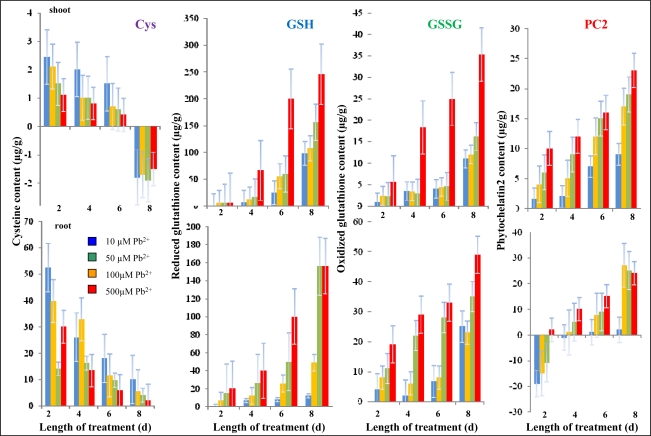
Changes of cysteine, GSH, GSSG and PC2 contents in sunflower plants exposed to Pb-EDTA. All data were obtained by subtraction from control plants. The experiment was carried out in triplicate.

Low molecular mass compounds rich in cysteine moieties play a very important role in the ability to withstand or even hyperaccumulate heavy metals ions. Due to this, we paid attention to such compounds. Particularly, the contents of cysteine, reduced glutathione (GSH), oxidized glutathione (GSSG) and PC2 were determined by high performance liquid chromatography with electrochemical detection (Figure 7). Contents of cysteine differed markedly in shoots and roots. Cysteine content declined in the roots of plants as the time of the treatment of plants with Pb-EDTA and concentration of toxic substance increased. Moreover, we observed ten times higher content of cysteine in roots in comparison with shoots. The observed reduction of cysteine content probably relates to its utilization for the biosynthesis of GSH and phytochelatins. Content of GSH was similar in roots and shoots and increased with increasing time of the treatment and concentration of Pb-EDTA (Figure 7). We plotted the dependence with linear regression to estimate the rate of synthesis of GSH. The rate expressed as the slope of the linear equation was 0.531*x* and 0.635*x* for roots and shoots, respectively, where “x” is concentration of applied Pb-EDTA.

It is a common knowledge that heavy metals induce generation of free oxygen species, which subsequently damage cell compartments (membranes, nucleic acids). Oxygen radicals can be scavenged by various mechanisms inside a cell [52]. Low molecular mass thiols are able to react with oxygen radicals via formation of disulphides. One of the most studied and well known reactions of such type is the redox cycling of GSH into GSSG [53]. In our experiment, GSSG levels gradually increased (Figure 7). We observed oxidative stress caused by Pb-EDTA in roots when the GSSG/GSH ratio was about 0.66. In shoots, the oxidative stress was less distinctive, with a GSSG/GSH ratio of 0.14. It follows from the results obtained that only a small part of the up-taken lead(II) ions is transported into shoots (stems, leaves). The content of PC2 in roots and shoots is shown in Figure 7. With increasing time of the treatment and concentration of Pb-EDTA the content was markedly enhanced. Moreover, the ability of plants to withstand oxidative stress caused by heavy metal depends also on rate of phytochelatin biosynthesis. Therefore, we again estimated the rate of phytochelatin biosynthesis via the slope of the linear equations plotted with data measured in each particular experimental group. The highest rate was detected in roots treated with 100 μM of Pb-EDTA (11.200) followed by 50 μM (13.270), 500 μM (7.100) and 10 μM (6.500). Compared to roots, the rate of phytochelatin biosynthesis was lower in shoots. The highest rate was detected in shoots treated with 50 μM of Pb-EDTA (4.600) followed by 100 μM (4.500), 500 μM (4.300) and 10 μM (2.719). It can be concluded that protective metabolic pathways of plants treated with 50 and 100 μM of Pb-EDTA was stimulated to withstand the adverse effect of heavy metal ions.

### 2.5. Monitoring of lead and magnesium distribution by LIBS

**Figure 8.**
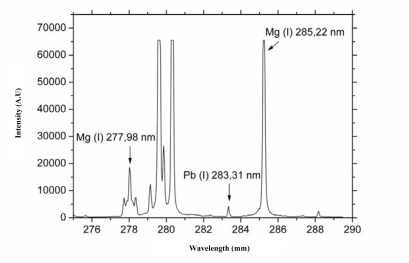
Typical LIBS spectrum after application of one laser pulse to a sample of common sunflower leaf exposed to 0.5 mM Pb-EDTA for 3 days.

To determine heavy metal ions many analytical instruments can be used, however, most of them are only able to quantify total contents of the metals [38,54–60]. This problem can be overcome using LIBS, because it is able to provide high spatial-distribution of metal ions in different types of materials [19,20,22,42,61,62]. Recently, we published the first experimental data focused on utilization of the LIBS technique for analysis of biological samples exposed to various heavy metals. It was demonstrated that the mentioned techniques are a unique analytical tool that can provide biologically interesting data [61,62]. The laser-generated patterns consisting of precisely ablated micro-craters have been utilized for mapping the lead and magnesium distribution on 4.5 × 2 mm^2^ leaf sections of sunflower samples. Measurement of optical emission of atoms by ICCD camera was carried out under optimized conditions. A typical LIBS spectrum after application of one laser pulse is shown in Figure 8.

Pb and Mg lines were identified in the measured spectral range. The analytical line of Pb (I) at 283.31 nm was used for detection of Pb (I). From the emission lines of magnesium, the 277.98 nm Mg (I) analytical line was selected. It was not possible to use other magnesium emission lines due to detector saturation. Subtraction of background was realized and the area under the emission line was calculated for all measurements.

**Figure 9.**
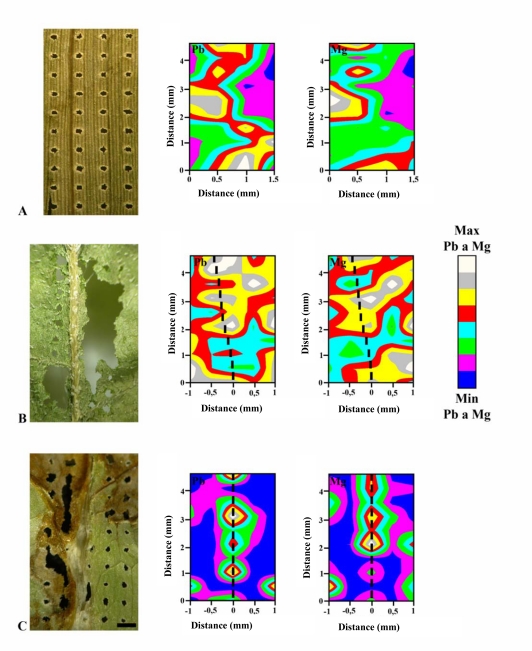
Spatial distribution of lead and magnesium in plant leaves: A – maize (*Zea mays*), B – sunflower (*Helianthus annuus*) and C – lettuce (*Lactuca sativa*) exposed to 0.5 mM Pb-EDTA for 5 days (maize, lettuce) and 3 days (sunflower). Ration scale represents 500 μm, vascular bundles are identified by lines. These plants were exposed to 0.5 mM Pb-EDTA for five days.

The data were used to assemble 3D Pb and Mg distribution maps. The Pb and Mg distribution in samples of maize leaf was relatively homogenous (Figure 9A). The three-dimensional distribution of Pb and Mg obtained by ablation of sunflower leaf samples exposed to 0.5 mM Pb-EDTA and the ablative patterns are shown in Figure 9B. The midrib is clearly evident in the sample due to distribution of Pb in this area. The distribution of Mg ions in this sample is homogenous, like in maize leaves. In the case of leaf samples of lettuce plant exposed to 0.5 mM Pb-EDTA, the highest content of both elements was determined in the midrib. This fact is clearly shown in Figure 9C. We also investigated the distribution of Pb and Mg in leaf samples of the same plants treated with 1 mM Pb-EDTA (3D maps on the right side in Figure 9). In these samples, we can see a heterogeneous distribution of Mg ions as well as Pb ions. Distribution of these elements is concentrated around the main vascular bundle of leaf, which means around the midrib. In all samples, LIBS measurements were compared with those obtained by laser ablation inductively coupled plasma mass spectrometry [41,42,63]. The results obtained were in good agreement. In the view using LIBS for monitoring of element accumulation in plant materials, the instrumentation is supplemented by software, which enables fully automated measurement [46].

## 3. Material and Methods

### 3.1. Chemicals

Acetonitrile and methanol (HPLC purity) were purchased from Merck (Darmstadt, Germany). Urease EC 3.5.1.5 (Jack Beans, type III; 45,000 IU/g) was purchased from Sigma Aldrich (St. Louis, MI, USA). Standards PC_2_, PC_5_ and DesGlyPC with purity higher than 90% were synthesized at Clonestar Biotech (Brno, Czech Republic). All other used chemical were also purchased from Sigma Aldrich, unless noted otherwise. The standard stock solutions (100 μg/mL) were prepared in ACS water (ie, chemicals that meet the specifications of the American Chemical Society) and stored in dark at 4 °C.

### 3.2. Cultivation of plants and sample preparation

Sunflower plants (*Helianthus annuus* L., Compositae) were used in our experiments. Sunflower kernels were germinated in the dark on wet filter paper in special vessels at 23 ± 2 °C. After ten days, maize seedlings were placed into vessels containing distilled water and cultivated in a Versatile Environmental Test Chamber (MLR-350 H, Sanyo, Japan) for eight days with 14 h daylight per day (maximal light intensity was about 100 μE/m^2^s^1^) at a temperature 23.5–25 °C and humidity 71–78%. After that, Pb-EDTA was added to the cultivation solution at final concentrations of 0, 10, 50, 100 and 500 μM. Plants grown without Pb-EDTA were used as a control. The sunflower plants placed in the vessels that distilled water with addition of Pb-EDTA (0, 10, 50, 100 and 500 μM) were grown for six days. Four plants each were harvested at certain time intervals (2^nd^, 4^th^, 6^th^ and 8^th^ day of the experiments), and their roots were rinsed three times in distilled water and 0.5 M EDTA. In addition, each harvested plant was divided into shoots (aerial plant parts) and roots. Fresh weight of the samples was measured immediately after the rinsing by using a Sartorius scale.

### 3.3. Sample preparation for thiol determination

Weighed plant tissues (approximately 0.2 g) were transferred to a test-tube, and liquid nitrogen was added. The samples were frozen to disrupt the cells [48]. The frozen sample was transferred to mortar and ground for 1 min. Then, 1,000 μL of 0.2 M phosphate buffer (pH 7.2) was added to the mortar, and the sample was grinding for 5 min. The homogenate was transferred to a new test-tube. The mixture was homogenised by shaking on a Vortex–2 Genie (Scientific Industries, New York, USA) at 4 °C for 30 min. The homogenate was centrifuged (14,000 *g*) for 30 min at 4 °C using a Universal 32 R centrifuge (Hettich-Zentrifugen GmbH, Tuttlingen, Germany). Before the analysis the supernatant was filtered through a membrane filter (0.45 μm Nylon filter disk, Millipore, Billerica, MA, USA).

### 3.4. High performance liquid chromatography with electrochemical detection

The HPLC-ED system consisted of two solvent delivery pumps operating in the range of 0.001–9.999 mL·min^−1^ (Model 582 ESA Inc., Chelmsford, MA), a Metachem Polaris C18A reverse-phase column (150 × 4.6; 3 μm particle size, Varian Inc., CA, USA) and a CoulArray electrochemical detector (Model 5600A, ESA). The electrochemical detector includes three flow cells (Model 6210, ESA, USA). Each cell consists of four analytical cells. One cell contains the working carbon porous electrode, two auxiliary and two reference electrodes. Both the detector and the reaction coil/column were thermostatted. The sample (10 μL) was injected using autosampler (Model 540 Microtiter HPLC, ESA, USA). For other experimental conditions see [24,28].

### 3.5. Automated spectrometric measurements

Spectrometric measurements were carried using an automated chemical analyser BS-200 (Mindray, China). Reagents and samples were placed on cooled sample holder (4 °C) and automatically pipetted directly into plastic cuvettes. Incubation proceeded at 37 °C. The mixture was consequently stirred. The washing steps with distilled water (18 mΩ) were done in the midst of the pipetting. The apparatus was operated using the BS-200 software (Mindray, China).

#### Urease activity determination – indophenol assay (Berthelot method)

Plant tissues samples (approximately 2 g) were homogenized in mortar for five minutes. Then twenty millilitres of 30% ethanol was added and this solution was poured into a bottle (50 mL) and vortexed at 300 rpm, 8 °C for 30 minutes using a vortexer (GFL, Germany). The extract was centrifuged for 10 min at 5,000 g (Hettich, Germany) and then the supernatant was collected. The supernatant (10 μL) was mixed with 448 μL of hypochlorite solution (12% NaOCl, 0.4 M Na_2_HPO_4_ and 0.37 M NaOH, adjusted to pH 12) and with 42 μL of phenol solution (sodium nitroprusside, 7% phenol). This mixture was stirred and incubated for 15 min at 37 °C. After this incubation the differences of absorption at 630 and 670 nm were measured [47,64].

#### ALT and AST activity determination

For standardization of determination of ATL and AST, sodium pyruvate (2 mM) in the concentration range 0–1.25 μkat/L was used. Into a test-tube containing 100 μL of sample or standard, 250 μL of substrate (for ALT dl-alanine 0.2 M, 2-oxoglutarate 2 mM, 0.1 M phosphate buffer pH 7.4; for AST l-aspartate 0.1 M, 2-oxoglutarate 2 mM, 0.1 M phosphate buffer pH 7.4) were added and this mixture was incubated for 60 min at 37°C in a thermostatted box. After 30 min the test-tubes were taken out the box and analysed using automated analyzer. The incubated solution (45 μL) was added to 45 μL of solution containing 2,4-dinitrophenylhydrazine (1 mM in 1 M HCl). This mixture was stirred and incubated for 10 min at 37 °C. Further, 180 μL of sodium hydrate (0.4 M) was added and newly stirred. Absorbance was measured after 10 min. at wavelength 530 nm.

#### Bradford protein assay

To 10 μL of sample 190 μL of Bradford reagent was added [65]. After 5 min of incubation at room temperature absorbance was measured at 595 nm against a blank sample (10 μL phosphate buffer and 190 μL Bradford reagent). Bradford reagent consists of 100 mg Coomassie Brilliant Blue G250, 50 mL 96% ethanol (*v*/*v*), 1,000 mL 8.5% phosphoric acid (*v*/*v*), 200 mL 0.1 M phosphate buffer pH 7.6). Total protein concentration was determined from calibration curve prepared by dilution of bovine serum albumin solution with the phosphate buffer within the concentration range from 0.05 to 1 mg/mL.

### 3.6. Laser induced breakdown spectroscopy

To realize the measurements with high-spatial resolution, the sample holder with the investigated species was placed to the stage with precision movements (2 μm in *x*, *y* and *z* direction) inside the ablation chamber (Tescan, Czech Republic). The single-shot LIBS analysis was performed in air under atmospheric pressure. The ablation spot was targeted and controlled for each shot by a CCD camera placed outside of the chamber. The LIBS micro-plasma was created using the second harmonic (532 nm) of a Nd:YAG laser system (Quantel, Brilliant B). The laser pulse width was ∼5 ns and the beam diameter 8 mm. The energy of the laser pulse was 10 mJ (at the sample). The laser-induced plasma was produced by focusing the laser beam with a 30 mm focal-length glass doublet (Sill Optics). Imaging system consisting of two quartz objectives was used to collect the LIBS micro-plasma radiation. Subsequently, the radiation was transported by a 3 m fibre optic system onto the entrance slit of the 0.32 m monochromator (Jobin Yvon TRIAX 320). In this study the grating 2,400 g/mm of the monochromator and 50 μm entrance slit were used. The dispersed spectrum of the plasma radiation was detected by an ICCD camera (Jobin Yvon Horiba). The time-resolved measurements were realized triggering the camera by the Q-switch signal of the laser. The detector was gated 1 μs after the Q-switch signal and the observation window was 10 μs. The lead-content within the leaf was detected by monitoring the 283.31 nm Pb (I) line in the created micro-plasmas.

## 4. Conclusions

We have demonstrated the ability of a laser-ablation based analytical method (LIBS) to map the distribution of lead and magnesium in the leaves of sunflower plants. Moreover, we have shown that the combination of LIBS with other precise analytical techniques such as high performance liquid chromatography with electrochemical detection and automated spectrometric analysis can provide many interesting results.

## References

[1] Macek T., Kotrba P., Svatos A., Novakova M., Demnerova K., Mackova M. (2008). Novel roles for genetically modified plants in environmental protection. Trends Biotechnol.

[2] Novakova M., Mackova M., Sylvestre M., Macek T. (2007). Preparation of genetically modified plants containing bacterial dioxygenase – Tool for preferable phytoremediation. J. Biotechnol.

[3] Najmanova J., Mackova M., Macek T., Kotrba P. (2007). Preparation of transgenic flax with enhanced metal tolerance. J. Biotechnol.

[4] Pavlikova D., Macek T., Mackova M., Sura M., Szakova J., Tlustos P. (2004). The evaluation of cadmium, zinc and nickel accumulation ability of transgenic tobacco bearing different transgenes. Plant Soil Environ.

[5] Pavlikova D., Macek T., Mackova M., Szakova J., Balik J. (2004). Cadmium tolerance and accumulation in transgenic tobacco plants with a yeast metallothionein combined with a polyhistidine tail. Int. Biodeterior. Biodegrad.

[6] Macek T., Mackova M., Pavlikova D., Szakova J., Truksa M., Cundy S., Kotrba P., Yancey N., Scouten W.H. (2002). Accumulation of cadmium by transgenic tobacco. Acta Biotechnol.

[7] Francova K., Macek T., Demnerova K., Mackova M. (2001). Transgenic plants – A potential tool for decontamination of environmental pollutants. Chem. Listy.

[8] Garbisu C., Alkorta I. (2001). Phytoextraction: a cost-effective plant-based technology for the removal of metals from the environment. Bioresour. Technol.

[9] Salt D.E., Blaylock M., Kumar N., Dushenkov V., Ensley B.D., Chet I., Raskin I. (1995). Phytoremediation – A novel strategy for the removal of toxic metals from the environment using plants. Bio-Technology.

[10] Fernandes J.C., Henriques F.S. (1991). Biochemical, physiological, and structural effects of excess copper in plants. Bot. Rev.

[11] Li X.D., Poon C.S., Liu P.S. (2001). Heavy metal contamination of urban soils and street dusts in Hong Kong. Appl. Geochem.

[12] Little P., Martin M.H. (1974). Biological monitoring of heavy-metal pollution. Environ. Pollut.

[13] Jarup L. (2003). Hazards of heavy metal contamination. Br. Med. Bull.

[14] Singh R.P., Tripathi R.D., Sinha S.K., Maheshwari R., Srivastava H.S. (1997). Response of higher plants to lead contaminated environment. Chemosphere.

[15] Sawidis T. (2008). Effect of cadmium on pollen germination and tube growth in Lilium longiflorum and Nicotiana tabacum. Protoplasma.

[16] Pandey S., Gupta K., Mukherjee A.K. (2007). Impact of cadmium and lead on Catharanthus roseus – A phytoremediation study. J. Environ. Biol.

[17] Doumett S., Lamperi L., Checchini L., Azzarello E., Mugnai S., Mancuso S., Petruzzelli G., Bubba M. (2008). Heavy metal distribution between contaminated soil and Paulownia tomentosa, in a pilot-scale assisted phytoremediation study: influence of different complexing agents. Chemosphere.

[18] Malkowski E., Kita A., Galas W., Karcz W., Kuperberg J.M. (2002). Lead distribution in corn seedlings (Zea mays L.) and its effect on growth and the concentrations of potassium and calcium. Plant Growth Regul.

[19] Kaiser J., Malina R., Galiova M., Novotny K., Diopan V., Adam V., Kizek R. (2007). Employment of laser spectrometry in heavy metal analysis. Lis. Cukrov. Repar.

[20] Stejskal K., Diopan V., Adam V., Zehnalek J., Trnkova L., Havel L., Galiova M., Malina R., Novotny K., Kaiser J., Kizek R. (2008). Study of effects of lead ions on sugar beet. Lis. Cukrov. Repar.

[21] Stejskal K., Supalkova V., Baloun J., Diopan V., Babula P., Adam V., Zehnalek J., Trnkova L., Havel L., Kizek R. (2007). Affecting of sugar beet (Beta vulgaris var. Altissima) by lead chelate. Lis. Cukrov. Repar.

[22] Krizkova S., Ryant P., Krystofova O., Adam V., Galiova M., Beklova M., Babula P., Kaiser J., Novotny K., Novotny J., Liska M., Malina R., Zehnalek J., Hubalek J., Havel L., Kizek R. (2008). Multi-instrumental analysis of tissues of sunflower plants treated with silver (I) ions – Plants as bioindicators of environmental pollution. Sensors.

[23] Supalkova V., Huska D., Diopan V., Hanustiak P., Zitka O., Stejskal K., Baloun J., Pikula J., Havel L., Zehnalek J., Adam V., Trnkova L., Beklova M., Kizek R. (2007). Electroanalysis of plant thiols. Sensors.

[24] Potesil D., Petrlova J., Adam V., Vacek J., Klejdus B., Zehnalek J., Trnkova L., Havel L., Kizek R. (2005). Simultaneous femtomole determination of cysteine, reduced and oxidized glutathione, and phytochelatin in maize (Zea mays L.) kernels using high-performance liquid chromatography with electrochemical detection. J. Chromatogr. A.

[25] Vacek J., Petrek J., Kizek R., Havel L., Klejdus B., Trnkova L., Jelen F. (2004). Electrochemical determination of lead and glutathione in a plant cell culture. Bioelectrochemistry.

[26] Petrek J., Baloun J., Vlasinova H., Havel L., Adam V., Vitecek J., Babula P., Kizek R. (2007). Image analysis and activity of intracellular esterases as new analytical tools for determination of growth and viability of embryonic cultures of spruce (Picea sp.) treated with cadmium. Chem. Listy.

[27] Zitka O., Stejskal K., Kleckerova A., Adam V., Beklova M., Horna A., Supalkova V., Havel L., Kizek R. (2007). Utilizing electrochemical techniques for detection of biological samples. Chem. Listy.

[28] Petrlova J., Mikelova R., Stejskal K., Kleckerova A., Zitka O., Petrek J., Havel L., Zehnalek J., Adam V., Trnkova L., Kizek R. (2006). Simultaneous determination of eight biologically active thiol compounds using gradient elution-liquid chromatography with Coul-Array detection. J. Sep. Sci.

[29] Supalkova V., Petrek J., Baloun J., Adam V., Bartusek K., Trnkova L., Beklova M., Diopan V., Havel L., Kizek R. (2007). Multi-instrumental investigation of affecting of early somatic embryos of spruce by cadmium (II) and lead (II) ions. Sensors.

[30] Ryant P., Dolezelova E., Fabrik I., Baloun J., Adam V., Babula P., Kizek R. (2008). Electrochemical determination of low molecular mass thiols content in potatoes (Solanum tuberosum) cultivated in the presence of various sulphur forms and infected by late blight (Phytophora infestans). Sensors.

[31] Lima P.R., Santos W.J.R., Oliveira A.B., Goulart M.O., Kubota L.T. (2008). Electrocatalytic activity of 4-nitrophthalonitrile-modified electrode for the L-glutathione detection. J. Pharm. Biomed. Anal.

[32] Gutscher M., Pauleau A.L., Marty L., Brach T., Wabnitz G.H., Samstag Y., Meyer A.J., Dick T.P. (2008). Real-time imaging of the intracellular glutathione redox potential. Nat. Methods.

[33] Timur S., Odaci D., Dincer A., Zihnioglu F., Telefoncu A. (2008). Biosensing approach for glutathione detection using glutathione reductase and sulfhydryl oxidase bienzymatic system. Talanta.

[34] Korn M.D.A., de Andrade J.B., de Jesus D.S., Lemos V.A., Bandeira M., dos Santos W.N.L., Bezerra M.A., Amorim F.A.C., Souza A.S., Ferreira S.L.C. (2006). Separation and preconcentration procedures for the determination of lead using spectrometric techniques: a review. Talanta.

[35] Korn M.D.A., dos Santos D.S.S., Welz B., Vale M.G.R., Teixeira A.P., Lima D.D., Ferreira S.L.C. (2007). Atomic spectrometric methods for the determination of metals and metalloids in automotive fuels – a review. Talanta.

[36] Lin T.J., Chung M.F. (2008). Using monoclonal antibody to determine lead ions with a localized surface plasmon resonance fiber-optic biosensor. Sensors.

[37] Shaw M.J., Haddad P.R. (2004). The determination of trace metal pollutants in enviromental matrices using ion chromatography. Environ. Int.

[38] Yantasee W., Lin Y., Hongsirikarn K., Fryxell G.E., Addleman R., Timchalk C. (2007). Electrochemical sensors for the detection of lead and other toxic heavy metals: the next generation of personal exposure biomonitors. Environ. Health Perspect.

[39] Janssens K.H.A., Adams F.C.V., Rindby A. (2000). X-ray fluorescence analysis.

[40] Jorks S. (1987). X-ray microscopy. Instrumentation and biological application.

[41] Kaiser J., Reale L., Ritucci A., Tomassetti G., Poma A., Spano L., Tucci A., Flora F., Lai A., Faenov A., Pikuz T., Mancini L., Tromba G., Zanini F. (2005). Mapping of the metal intake in plants by large-field X-ray microradiography and preliminary feasibility studies in microtomography. Eur. Phys. J. D.

[42] Kaiser J., Samek O., Reale L., Liska M., Malina R., Ritucci A., Poma A., Tucci A., Flora F., Lai A., Mancini L., Tromba G., Zanini F., Faenov A., Pikuz T., Cinque G. (2007). Monitoring of the heavy-metal hyperaccumulation in vegetal tissues by X-ray radiography and by femto-second laser induced breakdown spectroscopy. Microsc. Res. Tech.

[43] Becker J.S., Su J., Zoriya M.V., Dobrowolska J., Matusch A. (2007). Imaging mass spectrometry in biological tissues by laser ablation inductively coupled plasma mass spectrometry. Eur. J. Mass Spectrom.

[44] DeLucia F.C., Samuels A.C., Harmon R.S., Walters R.A., McNesby K.L., LaPointe A., Winkel R.J., Miziolek A.W. (2005). Laser-induced breakdown spectroscopy (LIBS): a promising versatile chemical sensor technology for hazardous material detection. IEEE Sens. J.

[45] Martin M.Z., Wullschleger S.D., Garten C.T., Palumbo A.V. (2003). Laser-induced breakdown spectroscopy for the environmental determination of total carbon and nitrogen in soils. Appl. Optics.

[46] Russo R.E., Mao X.L., Gonzalez J.J., Mao S.S. (2002). Femtosecond laser ablation ICP-MS. J. Anal. At. Spectrom.

[47] Hubalek J., Hradecky J., Adam V., Krystofova O., Huska D., Masarik M., Trnkova L., Horna A., Klosova K., Adamek M., Zehnalek J., Kizek R. (2007). Spectrometric and voltammetric analysis of urease - nickel nanoelectrode as an electrochemical sensor. Sensors.

[48] Petrek J., Vitecek J., Vlasinova H., Kizek R., Kramer K.J., Adam V., Klejdus B., Havel L. (2005). Application of computer imaging, stripping voltammetry and mass spectrometry to study the effect of lead (Pb-EDTA) on the growth and viability of early somatic embryos of Norway spruce (Picea abies/L./Karst.). Anal. Bioanal. Chem.

[49] Vitecek J., Petrlova J., Petrek J., Adam V., Havel L., Kramer K.J., Kizek R. (2007). Application of fluorimetric analysis of plant esterases to study of programmed cell death and effects of cadmium (II) ions. Biol. Plant.

[50] Vitecek J., Adam V., Petrek J., Vacek J., Kizek R., Havel L. (2004). Esterases as a marker for the growth of BY-2 tobacco cells and early somatic embryos of the norway spruce. Plant. Cell. Tiss. Org.

[51] Vitecek J., Petrlova J., Adam V., Havel L., Kramer K.J., Babula P., Kizek R. (2007). A fluorimetric sensor for detection of one living cell. Sensors.

[52] Droge W. (2002). Free radicals in the physiological control of cell function. Physiol. Rev.

[53] Noctor G., Foyer C.H. (1998). Ascorbate and glutathione: keeping active oxygen under control. Annu. Rev. Plant Physiol. Plant Molec. Biol.

[54] Adam V., Zehnalek J., Petrlova J., Potesil D., Sures B., Trnkova L., Jelen F., Vitecek J., Kizek R. (2005). Phytochelatin modified electrode surface as a sensitive heavy metal ion biosensor. Sensors.

[55] Adam V., Petrlova J., Potesil D., Zehnalek J., Sures B., Trnkova L., Jelen F., Kizek R. (2005). Study of metallothionein modified electrode surface behaviour in the presence of heavy metal ions-biosensor. Electroanalysis.

[56] Adam V., Hanustiak P., Krizkova S., Beklova M., Zehnalek J., Trnkova L., Horna A., Sures B., Kizek R. (2007). Palladium biosensor. Electroanalysis.

[57] Das A.K., de la Guardia M., Cervera M.L. (2001). Literature survey of on-line elemental speciation in aqueous solutions. Talanta.

[58] Rizk N.M.H., Abbas S.S., Hamza S.M., El-Karem Y.M.A. (2009). Thiopental and phenytoin as novel ionophores for potentiometric determination of lead (II) ions. Sensors.

[59] Bondarenko O., Rolova T., Kahru A., Ivask A. (2008). Bioavailability of Cd, Zn and Hg in soil to nine recombinant luminescent metal sensor bacteria. Sensors.

[60] Prasek J., Adamek M., Hubalek J., Adam V., Trnkova L., Kizek R. (2006). New hydrodynamic electrochemical arrangement for cadmium ions detection using thick-film chemical sensor electrodes. Sensors.

[61] Galiova M., Kaiser J., Novotny K., Novotny J., Vaculovic T., Liska M., Malina R., Stejskal K., Adam V., Kizek R. (2008). Investigation of heavy-metal accumulation in selected plant samples using laser induced breakdown spectroscopy and laser ablation inductively coupled plasma mass spectrometry. Appl. Phys. A-Mater. Sci. Process.

[62] Kaiser J., Galiova M., Novotny K., Cervenka R., Reale L., Novotny J., Liska M., Samek O., Kanicky V., Hrdlicka A., Stejskal K., Adam V., Kizek R. (2009). Mapping of lead, magnesium and copper accumulation in plant tissues by Laser-Induced Breakdown Spectroscopy and Laser-Ablation Inductively Coupled Plasma Mass Spectrometry. Spectrochim. Acta, Part B.

[63] Kaiser J., Galiova M., Novotny K., Reale L., Stejskal K., Samek O., Malina R., Palenikova K., Adam V., Kizek R. (2007). Utilization of the Laser Induced Plasma Spectroscopy for monitoring of the metal accumulation in plant tissues with high spatial resolution.

[64] Witte C.P., Medina-Escobar N. (2001). In-gel detection of urease with nitroblue tetrazolium and quantification of the enzyme from different crop plants using the indophenol reaction. Anal. Biochem.

[65] Bradford M.M. (1976). Rapid and sensitive method for quantitation of microgram quantities of protein utilizing principle of protein-dye binding. Anal. Biochem.

